# The role of nitric oxide, insulin resistance, and vitamin D in cognitive function of older adults

**DOI:** 10.1038/s41598-024-81551-3

**Published:** 2024-12-03

**Authors:** Samaneh Nakhaee, Rasul Azadi, Hamid Salehinia, Mitra Moodi, Asghar Zarban, Farshad Sharifi, Masoumeh Khorashadizadeh, Khadijeh Farrokhfall

**Affiliations:** 1 Medical Toxicology and Drug Abuse Research Center (MTDRC), Birjand University of Medical Sciences, Birjand, Iran; 2 Geriatric Health Research Center, Birjand University of Medical Sciences, Birjand, Iran; 3 Social Determinants of Health Research Center, Birjand University of Medical Sciences, Birjand, Iran; 4 Cardiovascular Diseases Research Center, Birjand University of Medical Sciences, Birjand, Iran; 5 Elderly Health Research Center, Endocrinology and Metabolism Population Sciences Institute, Tehran University of Medical Sciences, Tehran, Iran

**Keywords:** Nitric oxide, MMSE, Cognition impairment, Insulin resistance, Neuroscience, Diseases, Medical research, Neurology

## Abstract

Aging is accompanied by possible cognitive impairment, as well as a reduction in nitric oxide (NO) bioavailability, insulin resistance, and vitamin D deficiency. Among these Nitric oxide exhibits a dual role in brain biology, where both low and high levels can have detrimental effects on memory and neurotoxicity. In this study we aimed to investigate the role of nitric oxide, insulin resistance, and vitamin D in cognitive function of elderly individuals. This case-control study involved participants aged ≥ 60 years who were under observation in the Birjand Longitudinal Aging Study. A total of 40 participants were selected as controls, while 64 participants with cognitive impairment were identified as cases. The cases were further subdivided according to their Mini-Mental State Examination (MMSE) scores into two groups: mild and moderate cognitive impairment. Cognitive function in both cases and controls was assessed using the MMSE. The levels of total NOx, vitamin D, insulin, and blood glucose were measured using ELISA and spectrophotometric immunoassays. Insulin resistance indices, such as homeostasis model assessment of insulin resistance (HOMA-IR) and quantitative insulin sensitivity check index (QUICKI) were calculated. Serum NOx levels were significantly lower in participants with cognitive impairment compared to the control group (*p* < 0.01). However, the mean (SD) levels of serum vitamin D, insulin, and glucose, as well as the insulin resistance markers, showed no significant differences between the groups. A positive correlation was observed between serum NOx levels and MMSE scores. NO may be an essential factor for normal brain function, as serum NOx levels were significantly higher in controls compared to those with cognitive impairment, and there was a positive correlation with MMSE scores. Other metabolic and nutritional markers, including vitamin D and insulin resistance, did not demonstrate a significant effect.

## Introduction

Aging is a continuous, detrimental, and inherent process for all living creatures^1^. By the year 2030, one out of every six people worldwide will be 60 years or older, resulting in an increase in this age group’s population from 1 billion in 2020 to 1.4 billion^2^. At least 20% of adults aged 60 and older have mental or neurological disorders (excluding headache disorders)^2^. Dementia and depression rank among the most prevalent neurological and mental disorders, affecting nearly 5 to 7% of the world’s older population^3^. The cognitive decline associated with aging appears to be a continuous process, with some individuals experiencing accelerations. Each person differs in the pace, shape, and rate of abrupt cognitive declines^4^.

Aging is closely associated with a decline in insulin action^5,6^. Age-related insulin resistance (IR) is characterized by decreased sensitivity of tissues especially muscle, adipose tissue, the brain, and other organs to insulin. IR can lead to cerebral hypoperfusion and the accumulation of amyloid beta-protein (Aβ). In this situation, the clearance of Aβ is reduced, increasing the risk of cognitive impairment^6^. Decreased activity of the phosphatidylinositol 3-kinase (PI3-K)/Akt pathway, along with heightened expression of mitogen-activated protein kinase pathways, which are characteristic of IR, disrupts the equilibrium between these signaling pathways. This disruption leads to endothelial dysfunction, decreased nitric oxide (NO) production within endothelial cells, and initiates an inflammatory cascade, oxidative stress, and advancing cognitive impairment^5,6^.

It has been suggested that oxidative stress and vascular dysfunction play a role in the pathogenesis of both Alzheimer’s disease (AD) and vascular dementia^7^. NO is a free radical synthesized by a family of nitric oxide synthases (NOS), which includes constitutive neuronal NOS (nNOS), inducible NOS (iNOS), and endothelial NOS (eNOS)^7^. Physiologically, NO is a fundamental gas molecule crucial for the vasodilation of cerebral and peripheral vessels and plays a significant role in cognitive function as an intercellular messenger^8,9^. Pathologically, however, NO is considered a strong oxidant and an active free radical that can react with superoxide anions (O₂⁻) to form peroxynitrite (ONOO⁻). As an oxidizing agent, peroxynitrite creates an environment that damages susceptible macromolecules, such as proteins through degeneration and deactivation, lipid peroxidation, and DNA damage, ultimately resulting in cell damage and mediating neurodegeneration in neurological disorders.

At high levels, nitric oxide (NO) produced in microglia is neurotoxic and may play a role in neurodegeneration. It is cytotoxic to cancer cells and invading microorganisms, as well as damaging to the tissues that generate it^10^. NO facilitates neural damage through oxidative stress, energy depletion, inhibition of DNA synthesis, DNA damage, and the induction of programmed cell death^11^. Studies on NO levels and cognition have yielded contradictory results. It has been shown that increasing levels of NO are associated with cognitive impairments in Alzheimer’s disease (AD) patients, and even in animal studies, NO has been linked to aging, memory loss, and dementia^8,9^. However, one study concluded that cognitive impairment does not correlate with the levels of cerebrospinal fluid (CSF) or serum NO^10^. Previous research has reported changes in CSF^12^ and plasma nitrate levels in AD patients^13,14^, but the hypothesis of a relationship between NO and cognitive function has not yet been fully understood.

Another factor with neuroprotective effects that promotes brain health is vitamin D (Vit D) (active form: 25(OH)D)^15,16^, and the elderly may experience vitamin D deficiency^17^. Vit D indicates its function by upregulating the gene expression of various proteins required for synapse growth and neurogenesis, especially in the hippocampal region^17^, as well as the gene expression of neurotransmitters such as acetylcholine^18^. Furthermore, vitamin D reduces hallmark age-related pathologies in the brain^18^. Additionally, sufficient serum levels of Vit D have been associated with total hippocampal volume^19^.

Pathological mechanisms of central nervous system dysfunction in most neurological disorders remain obscure. The relationship between serum nitric oxide levels and cognitive function has been explored in previous studies, primarily focusing on patients with AD and dementia^7,10,11,14^. However, there is a notable gap in the literature regarding the assessment of cognitive impairment specifically in elderly people without these diagnoses. In this study, we focus on cognitive impairment in older adults, which encompasses a range of conditions that is not limited to AD and dementia. Specifically, this study is directed at the assessment of cognitive impairment in older adults with different degrees of cognitive impairment as classified by the MMSE scores. Our study tries to fill this gap by investigating the associations between serum NOx levels, insulin resistance parameters, and vitamin D levels in older adults with varying degrees of cognitive impairment compared to a healthy old group. The study of these combined factors is an attempt at making an additional contribution to the understanding of how such elements act upon the cognitive health of the aging population. Considering the prevalence of insulin resistance in aging and its cognitive significance, along with the role of the nitric oxide pathway in the development of Alzheimer’s disease, this study aims to investigate insulin resistance, serum levels of nitric oxide, and vitamin D in healthy and cognitively impaired elderly individuals.

## Materials and methods

The current case-control study was conducted within the Birjand Longitudinal Aging Study (BLAS) among elderly subjects aged 60 years or above, both in urban and rural settings of the city of Birjand, South Khorasan Province, Iran. In this case-control study participants with cognitive impairment (case group) were compared with those without cognitive impairment (control group) to assess the association between serum nitric oxide levels, insulin resistance, vitamin D levels, and cognitive function. Informed consent was obtained from all participants and/or their legal guardians. Based on the study by Bosco et al.^20^, which considered the means of two groups as 4.2 and 2.5, respectively, and the standard deviations as 1.5 and 1, with 90% power and 95% confidence limits, the sample size in each group consisted of 21 individuals. Taking into account a 10% attrition rate, the adjusted sample size was 23 individuals per group. To enhance the power and generalizability of the results, the sample size was doubled in both groups.

Inclusion criteria included individuals over 60 years of age. Exclusion criteria included individuals who were bedridden, with severe cognitive impairment, or those diagnosed with Alzheimer’s disease; inability to communicate, and less than six months of predictive life expectancy. Healthy controls were selected by excluding significant co-morbid conditions such as ischemic heart disease (IHD), hypertension (HTN), cerebrovascular accident (CVA), malignancy, endocrine disorders, and taking drugs affecting cognitive functions^21^. Persons with a body mass index (BMI) of 40 or above were excluded to make the groups comparable. Both groups underwent cognitive assessments, ensuring the control group did not exhibit cognitive impairments. Importantly, all participants in the control group had MMSE scores of 22–30, indicating normal cognitive function. Detailed inclusion and exclusion criteria are presented in Table 1. The cases and controls were matched based on age, sex, BMI, and blood pressure.

**Table 1:** The inclusion criteria considered for the case and control groups.

Inclusion criteria	
Control	Cognitive impairment
Aged ≥ 60 years old	Aged ≥ 60 years old
Residence in Birjand County	Residence in Birjand County
Willing to comply with the study protocol	Willing to comply with the study protocol
MMSE score ≥ 22	9 < MMSE score < 22
Any history of IHD, HTN, CVA, cancer, diabetes, and other endocrinopathy	Any history of IHD, HTN, CVA, cancer, diabetes, and other endocrinopathy
No taking any specific drugs	No taking any specific drugs
Absence of very severe cognitive impairment or Alzheimer’s disease	Absence of very severe cognitive impairment or Alzheimer’s disease
BMI < 40	BMI < 40

*MMSE* Mini-Mental State Examination, *BMI* Body Mass Index, *IHD* Ischaemic Heart Disease, *HTN* hypertension, *CVA* cerebral vascular accident.

Demographic data, including age and sex, along with cognitive evaluations, were collected using a questionnaire. Anthropometric measurements, including height and weight, were obtained, and BMI was calculated. Cognitive assessment data were gathered using the MMSE questionnaire, which has been validated for reliability and validity in the Iranian population^22,23^. Based on the MMSE, the maximum score is 30. In this study, participants with a score below 22 were included in the cognitive impairment (CI) group^22^. The CI group was further divided into two subgroups based on the MMSE score of the population: mild CI and moderate CI. The mild and moderate CI subgroups had MMSE scores of 19–22 and 9–18, respectively. After 12 h of fasting, 10 cc of blood was drawn between 8 and 10 AM. The blood serum was then separated and transferred to a -80 °C freezer. Plasma glucose, insulin, and total NOx levels were measured using laboratory tests. Insulin levels were assessed with an ELISA kit (Monobind Inc., Cat# 5825 − 300, USA) and detected by an automated machine (EUROIMMUN analyzer I-ZP, Germany). Glucose levels were determined using the glucose oxidase method with a fully automated Prestige 24i analyzer (Tokyo, Japan). The NOx level was estimated using the Griess reaction, which measures nitric oxide metabolites in the form of nitrite and nitrate (NOx) and is reported in µmol per liter of serum. Initially, samples were deproteinized with 90% ethanol by adding an equal volume of ethanol to the sample, then mixed vigorously and centrifuged at 10,000 G for 10 min at 4 °C. The clear supernatant was used to estimate nitric oxide metabolites. To measure total nitrite and nitrate (NOx), 100 µl of supernatant was combined with 100 µl of saturated vanadium chloride (III) solution (0.8% W/V) in a microplate to convert nitrate to nitrite. Following this, 50 µl of 0.2% sulfanilamide and 50 µl of 0.1% N-(1-naphthyl)-ethylenediamine dihydrochloride (NEDD) were added, and the mixture was incubated for 30 min at 37 °C. After the reaction was complete and color formation occurred, the color absorbance was read by an ELISA reader at 540 nm^24^. The concentration of samples was calculated using a calibration curve created with various concentrations of potassium nitrate. HOMA-IR (homeostasis model assessment of insulin resistance) and QUICKI (quantitative insulin sensitivity check index) were calculated based on fasting serum glucose and insulin levels. HOMA-IR serves as an index for insulin resistance, assessing the roles of insulin resistance and beta-cell dysfunction in hyperglycemia. The accuracy and precision of HOMA-IR are comparable to hyperglycemic/euglycemic clamps and intravenous glucose tolerance tests. The cut-off point is 1.77, with lower results considered normal in the Iranian population^25^. The insulin resistance index was measured as follows:

$$\:\text{H}\text{O}\text{M}\text{A}-\text{I}\text{R}=\frac{(\text{F}\text{a}\text{s}\text{t}\text{i}\text{n}\text{g}\:\text{G}\text{l}\text{u}\text{c}\text{o}\text{s}\text{e}\left(\frac{mmol}{l}\right)\times\:\text{F}\text{a}\text{s}\text{t}\text{i}\text{n}\text{g}\:\text{I}\text{n}\text{s}\text{u}\text{l}\text{i}\text{n}\left(\frac{\mu\:U}{ml}\right))}{22.5}$$

QUICKI is an index used to measure insulin sensitivity accurately and simply in clinical investigations. The highest normal values for Iranian men and women are cut-off points of 0.343 and 0.331, respectively^26^. The insulin sensitivity index was measured as follows:

$$\:\text{Q}\text{U}\text{I}\text{C}\text{K}\text{I}=\:\frac{1}{Log\:\text{F}\text{a}\text{s}\text{t}\text{i}\text{n}\text{g}\:\text{i}\text{n}\text{s}\text{u}\text{l}\text{i}\text{n}\:\left(\frac{\mu\:U}{ml}\right)+Log\:\text{f}\text{a}\text{s}\text{t}\text{i}\text{n}\text{g}\:\text{g}\text{l}\text{u}\text{c}\text{o}\text{s}\text{e}\:\left(\frac{mg}{dl}\right)}$$

### Statistical analysis

Data were analyzed using SPSS version 19, employing mean, standard deviation, and frequency distribution tables for data description. The normality of the data was assessed using the Kolmogorov-Smirnov test. A one-way ANOVA test was used to compare means between groups, while non-normally distributed data were compared using the Kruskal-Wallis test. Dunn’s multiple comparisons test was applied for pairwise comparisons of groups. The correlation between variables was assessed using the Spearman correlation test. A p-value of less than 0.05 (two-tailed) was considered statistically significant, and results were presented as mean ± standard deviation.

## Results

The descriptive characteristics of the analyzed groups are summarized in Table 2. Among all participants included in the study, there were 53 men and 51 women. The demographic, clinical, and anthropometric characteristics of the participants were not significantly different between the case and control groups (Table 2). The mean MMSE score was significantly higher in the control group compared to the CI groups (*p* < 0.001). Additionally, the mild CI group had a higher MMSE score than the moderate CI group (*p* < 0.001).

**Table 2:** Descriptive characteristics of the studied groups.

Variables	Control group (*n* = 40)	Mild CI (*n* = 32) (high median cut)	Moderate CI (*n* = 32) (low median cut)	*P*-value
Sex (male)	23 (57.5%)	14 (43.75%)	14 (43.75%)	0.32
Age (years)	68.18 ± 6.52	71.44 ± 7.26	69.19 ± 6.82	0.107
Weight (kg)	60.18 ± 11.34	55.72 ± 11.66	61.00 ± 13.02	0.16
Height (m)	159.6 ± 10.32	155.3 ± 9.66	156.2 ± 8.07	0.12
BMI	23.67 ± 3.91	23.11 ± 4.62	24.96 ± 4.49	0.22
Systolic BP (mmHg)	123.0 ± 12.41	119.7 ± 14.44	115.5 ± 16.4	0.36
Diastolic BP (mmHg)	74.79 ± 9.29	76.14 ± 9.16	79.3 ± 10.36	0.13
MMSE Score	27.03 ± 2.19	20.22 ± 1.03*	15.34 ± 2.26*^†^	< 0.001

Results are expressed as mean ± SD. Data on age, BMI, and MMSE score were not normally distributed and were analyzed using Kruskal-Wallis test and Dunn’s multiple comparisons test. The Chi-square test was employed for sex comparisons between groups. The means of the remaining variables were compared using one-way ANOVA.

*CI* cognitive impairment, *BP* blood pressure, *BMI* Body mass index, *MMSE* Mini-Mental State Examination.

*Vs control group.

^†^Vs mild cognition impairment.

The serum NOx level was significantly lower in the moderate CI group compared to the control group (*p* = 0.002) and was also significantly lower than in the mild CI group (*p* = 0.01). Serum insulin and vitamin D levels did not show significant differences between the groups. Furthermore, the HOMA-IR and QUICKI indices did not exhibit any significant differences between the cases and control groups (Table 3).

**Table 3:** The comparison of NOx, vit D levels, and carbohydrate metabolic parameters in the serum of studied groups.

Variables	Control group	Mild CI (high median cut)	Moderate CI (low median cut)	*P*-value
Serum NOx level (µmol/L)	144.6 ± 63.86	134.4 ± 51.55	94.62 ± 49.83*^†^	0.001
Serum Vit D (ɳg/mL)	30.54 ± 13.23	29.05 ± 12.25	33.32 ± 19.70	0.55
Serum insulin level (µIU/mL)	4.41 ± 2.44	4.44 ± 3.81	4.55 ± 4.25	0.52
HOMA-IR index	1.06 ± 0.61	1.12 ± 1.06	1.04 ± 1.06	0.38
QUICKI index	0.4 ± 0.05	0.41 ± 0.07	0.41 ± 0.06	0.43

Results are expressed as mean ± SD. Vit D levels were compared using one-way ANOVA. The remaining data were not normally distributed and were analyzed using the Kruskal-Wallis and Dunn’s multiple comparisons tests.

*CI* cognitive impairment, *QUICKI* quantitative insulin sensitivity check index, *HOMA-IR* homeostasis model assessment of insulin resistance.

*Vs Control group.

^†^Vs Mild CI.

Spearman’s correlation was used to determine the relationships between variables in the studied population (Table 4). There was a strong positive correlation between the MMSE score and serum NOx level (*r* = 0.329, *p* = 0.0008, *n* = 100), indicating that higher cognitive function is associated with more NOx levels in serum. The BMI was positively correlated with the HOMA-IR index (*r* = 0.38, *p* < 0.0001, *n* = 102), while it exhibited an inverse correlation with the QUICKI index, both of which were statistically significant (*r* = -0.367, *p* < 0.0001, *n* = 102).

**Table 4:** Correlation between variables in all participants.

	MMSE	NOx	HOMA-IR	QUICKI	Vit D	BMI
MMSE	1	0.329*	0.105	− 0.099	− 0.011	0.056
NOx		1	0.119	− 0.108	0.067	0.086
HOMA-IR			1	− 0.995*	0.022	0.38*
QUICKI				1	− 0.029	− 0.367*
Vit D					1	− 0.030
BMI						1

The data were not normally distributed and the correlations were calculated using Spearman’s correlation test.

*P < 0.001.

## Discussion

In this study, we investigated plasma levels of nitric oxide and insulin resistance in individuals with impaired cognition and in control groups. Our results showed that individuals with moderate cognitive impairment had lower serum NOx levels compared to those with mild cognitive impairment (MCI) and healthy individuals, whereas the HOMA-IR and QUICKI indices did not differ significantly between the studied groups. Our findings are consistent with the studies by Corzo et al.^7^ and Selly et al.^14^, which noted decreased serum levels of nitric oxide in various forms of dementia. Supporting these results, L-arginine, the precursor of NO^27^ and NO donors^28^ have been confirmed to have therapeutic effects in patients with age-related degenerative diseases such as Alzheimer’s disease (AD). NO can mediate neuroprotection by eradicating free radicals, such as superoxide, and elevating the levels of cytoprotective proteins. Long-term potentiation (LTP), the fundamental physiological mechanism of learning and memory formation^29,30^, is modulated by NO in glutamatergic neurons^31^. Furthermore, NO induces dendritic spine growth in both post-synaptic and pre-synaptic endings, which, in turn, improves memory capacity and storage^32^. NO is the primary molecule linking brain blood flow to neuronal activity, a physiological mechanism known as neurovascular coupling^33^. Reduced NO availability has a neurodegenerative effect due to neurovascular endothelial dysfunction, disruption of brain blood flow, and resultant cognitive impairment^34,35^. Venturelli et al. concluded that the progressive depletion of NO bioavailability and reduction in central blood circulation are directly associated with AD pathogenesis, primarily involving peripheral circulation^35^. The results of a clinical trial conducted by Wightman et al. showed that consuming a nitrate-rich diet resulted in significant improvements in cognitive activity due to increased brain circulation^36^, especially in the regional brain circulation of the white matter in the frontal and prefrontal lobes, as well as the anterior cingulate cortex^37^.

Studies on NO levels and aging have yielded conflicting results. One study concluded that nNOS activity increases as a compensatory response to decreased NMDA receptor activity during the aging process^38^. However, additional studies have confirmed that L-arginine-NO signaling declines with age. A longitudinal study demonstrated that NO concentration dynamics decreased in the hippocampus, accompanied by cognitive impairment in a model of physiological aging in rats^39^. Furthermore, nNOS activity^40^, nNOS gene expression, and glutamate receptor levels decreased with aging. NO bioactivity was dysregulated in brain aging, leading to the production of more reactive oxidants, such as ONOO−, which destroy signaling pathways and induce cell damage^41^. Changes in NO bioactivity are also evident in cognitive disorders, where aging is a significant risk factor^42^. Results from a proteomic study confirmed by immunohistochemistry and Western blot analysis showed a correlation between amyloid-beta deposition and the nitration of multiple proteins in the hippocampus of AD patients^43^. In contrast, another study concluded that cognitive impairment is not reflected in CSF or serum NO levels^10^. Neuronal hyperactivity is more pronounced in subjects with cognitive impairment^44,45^. Longitudinal study results indicated that hyperexcitability is followed by a more rapid decline in hippocampal activation, ultimately leading to significant loss of hippocampal activation and memory formation^46^. Neuronal hyperexcitability can be defined as an increased neuronal activation in response to stimuli, such as memory-encoding tasks^47^. Among the multiple factors contributing to neural hyperexcitability, impaired Ca²⁺ homeostasis and excessive glutamate release are the main underlying mechanisms modulated by NO. As mentioned, dyshomeostasis of NO is associated with cognitive impairment, with both excessive NO generation and reduced NO bioavailability implicated.

Although HOMA-IR and QUICKI indices were not substantially different between the two groups, results were higher in the case group. In a case-control study by Bosco et al. aimed at investigating the association between dementia and insulin resistance (IR) in patients with Parkinson’s disease (PD), it was shown that IR acts as an independent risk factor for impaired cognition in PD patients^20^. In patients with cerebral small vessel disease (CSVD), IR worsens vascular cognitive impairment (VCI), as demonstrated by Guo et al.^6^. In the pathology of AD, failure of insulin and IGF signaling pathways leads to aberrant tau phosphorylation, accumulation of neurofibrillary tangles, senile plaque formation (such as amyloid-beta precursor protein [AβPP] and Aβ peptides), mitochondrial dysfunction, and endoplasmic reticulum (ER) stress, which propagate cell death cascades and disrupt cholinergic homeostasis, ultimately impairing memory formation and cognition^48^. Our present study included healthy older subjects, whereas a recent cohort study indicated that insulin resistance predicts cognitive impairment primarily in prediabetic^49^ and, diabetic subjects^50^.

Experimental and cellular studies have attributed beneficial effects of vitamin D on memory formation and cognitive function, as well as its neuroprotective effects, including the clearance of amyloid plaques^51–53^. However, longitudinal clinical studies with large sample sizes over 12–20 years have shown a wide range of results, from no significant association between cognitive decline and serum vitamin D levels^54,55^ to poor performance based on MMSE scores in vitamin D deficiency^56^. It appears that cognitive impairment may lead to lower health and increased risk factors for vitamin D deficiency, rather than vitamin D deficiency causing cognitive impairment^57^.

Residual confounding is unavoidable in this study due to variables that were not measured or inadequately measured, such as dietary nitrate-nitrite and micronutrient supplementation intake among participants, genetic data, etc. Consequently, our study design cannot eliminate all potential confounding factors, and some variables may still have an impact. Additionally, the mechanism by which decreased NOx levels contribute to the development of dementia remains unknown and requires further investigation. We acknowledge that the relatively limited number of participants in our study could be a limitation to the generalization of our results. Although we identified possible associations between nitric oxide levels and cognitive impairment, the constrained number of subjects limits any firm conclusions. Larger and more diverse studies are therefore needed to confirm our findings and further explore the complex interactions between various metabolic and nutritional markers in relation to cognitive health. Another limitation of our study is the inability to measure serum cortisol levels, which could have provided additional insight into the relationship between stress, nitric oxide activity, and cognitive function. Future studies should consider cortisol assessment for a comprehensive comparison of results related to cognitive functioning.

## Conclusion

Based on the results of the present study serum NOx levels were significantly lower in elderly participants with cognitive impairment compared to the control group and serum NOx level had a positive correlation with the MMSE score. Although our study elicited that serum NOx level showed a positive correlation with MMSE scores, this does not necessarily imply a cause-and-effect relationship, but rather a possible association. Moreover, the other metabolic and nutritional markers did not have the same association, such as vitamin D and insulin resistance. These findings point out the potential of serum NOx as a biomarker for cognitive health in older adults. Though the other metabolic parameters did not show significant differences, which undoubtedly will have to be investigated further, our results suggest that targeting nitric oxide pathways may offer new avenues for clinical intervention in cognitive impairment. Further studies should be done to clarify the responsible mechanisms and evaluate the therapeutic potential of NOx supplementation in improving cognitive outcomes in older individuals.

## Data Availability

The authors declare that the raw data used for statistical analysis are available on the Birjand Longitudinal Aging Cohort Study (BLAS). The analyzed data are available within the paper in the form of tables. The remaining datasets used and/or analyzed during the current study are available from the corresponding author upon reasonable request with permission of the Birjand Longitudinal Aging Cohort Study (BLAS) administrator.
